# Applying Machine Learning to Finger Movements Using Electromyography and Visualization in Opensim

**DOI:** 10.3390/s22103737

**Published:** 2022-05-14

**Authors:** Jose Amezquita-Garcia, Miguel Bravo-Zanoguera, Felix F. Gonzalez-Navarro, Roberto Lopez-Avitia, M. A. Reyna

**Affiliations:** 1 Facultad de Ingeniería, Universidad Autónoma de Baja California, Mexicali 21280, Mexico; jose.amezquita@uabc.edu.mx (J.A.-G.); ravitia@uabc.edu.mx (R.L.-A.); 2 Ingeniería en Mecatrónica, Universidad Politécnica de Baja California, Mexicali 21376, Mexico; 3 Instituto de Ingeniería, Universidad Autónoma de Baja California, Mexicali 21280, Mexico; fernando.gonzalez@uabc.edu.mx (F.F.G.-N.); mreyna@uabc.edu.mx (M.A.R.)

**Keywords:** electromyography, classification model, biomechanical simulation

## Abstract

Electromyographic signals have been used with low-degree-of-freedom prostheses, and recently with multifunctional prostheses. Currently, they are also being used as inputs in the human–computer interface that controls interaction through hand gestures. Although there is a gap between academic publications on the control of an upper-limb prosthesis developed in laboratories and its service in the natural environment, there are attempts to achieve easier control using multiple muscle signals. This work contributes to this, using a database and biomechanical simulation software, both open access, to seek simplicity in the classifiers, anticipating their implementation in microcontrollers and their execution in real time. Fifteen predefined finger movements of the hand were identified using classic classifiers such as Bayes, linear and quadratic discriminant analysis. The idealized movements of the database were modeled with Opensim for visualization. Combinations of two preprocessing methods—the forward sequential selection method and the feature normalization method—were evaluated to increase the efficiency of these classifiers. The statistical methods of cross-validation, analysis of variance (ANOVA) and Duncan were used to validate the results. Furthermore, the classifier with the best recognition result was redesigned into a new feature space using the sparse matrix algorithm to improve it, and to determine which features can be eliminated without degrading the classification. The classifiers yielded promising results—the quadratic discriminant being the best, achieving an average recognition rate for each individual considered of 96.16%, and with 78.36% for the total sample group of the eight subjects, in an independent test dataset. The study ends with the visual analysis under Opensim of the classified movements, in which the usefulness of this simulation tool is appreciated by revealing the muscular participation, which can be useful during the design of a multifunctional prosthesis.

## 1. Introduction

Research in prosthetic hand control applications most often involves the decoupling of the surface electromyography (sEMG) signal to decipher the natural regulation. Interpretation of the sEMG signals is an active area of research. One objective is to achieve efficient control of prostheses, similar to the natural movements of the body. Within these research publications, one can find studies searching for the ideal place for the electrodes in the muscular region of interest [1,2,3,4]; studies focused on the importance of the type, size, shape and material of the electrode to be used [5,6,7,8,9]; studies using data mining to find features in the raw sEMG signals to achieve their decoding [10,11,12]; and studies evaluating the implementation to detect the target movements [13,14,15], aiming to implement the processing in systems embedded in microcontrollers.

Prosthetic electronic devices increasingly have more degrees of complexity; however, it is reported that, within these devices, 60% have 1 to 4 degrees of freedom (DoFs), 30% have 5 to 10 DoFs and only 10% have more than 10 DoFs [16]. A DoF within a prosthetic device should be understood as the motion in one direction of the possible movements of a natural joint. Devices and applications evolve into more complex systems; therefore, control systems with a greater number of functionalities are necessary, but they must be intuitive for human control, which is possible with machine learning techniques [15]. A considerable number of investigations work with machine learning for the classification and interpretation of EMG signals [17,18,19,20]; however, there is still no model that is applicable in complex systems and outside of controlled environments in laboratories. In our investigations regarding the decoding of the sEMG signal, we found the use of deep learning can be used as a method to improve classic classifiers but demands a large amount of data through many layers of processing [18], the reduction in dimensionality through specific methods [19] allows the selection of a smaller number of features with higher information quality, and the use of time–frequency features [20] allows extracting information hidden in the raw EMG signal and the transfer to new spaces to extract relevant information. Even though diverse research approaches and applications are emerging in the state of the art, such as the mathematically intense genetic algorithms and adaptive neuro-fuzzy systems [21,22] which are powerful methods to solve difficult regression problems, the load of monitoring the progress of each of the DoFs involved in the movement control would make it impractical with these models. The control of upper-limb prosthetic systems requires further research regarding practical utility, and here using traditional methods to classify multiple discrete targets brings an elegant and viable solution.

It is difficult to obtain real-time applications with multifunctional prostheses due to, among other issues, the nature of the sEMG signal and the large number of processing operations required to analyze it. Another drawback regarding progress is the limited availability of standardized EMG databases, but still, there are some databases that help address the complexity of multifunctionality and which have sEMG signal conditioning best practice elements. There are known sites for online multifunction EMG databases that include movements specific to the hand and fingers [23,24,25,26,27,28,29]. Some of the sites have more than one dataset with different features [24,25,28,29]. The amounts of electrodes and movements vary, but the movement experiments are captured with EMG surface electrodes, and some of the databases also contain information from other types of sensors.

In a previous work [17], a first attempt was made to create a simple classification model of EMG signals, to be combined with a modified virtual biomechanical model of the wrist and hand in Opensim [30]. In this work, we propose using an online database to decode the coordinated muscle activity obtained from an array of sEMG electrodes in the forearm, to develop a model that classifies 15 hand movements, and to transform the classification sequence into virtual movement with the Opensim environment. In Opensim, the evolution of muscle movements can be virtually observed from the results of the classification model and visually compared with those of the ideal movement.

## 2. Materials and Methods

### 2.1. Database of Predefined Finger Movements

Although the relatively poor repeatability of the surface EMG measures is a known issue [31], an exhaustive search was conducted to find databases published online that were open access [23,24,25,26,27,28,29], to determine which was the most convenient to use. A database found in an EMG repository was selected [25] since it was one of the most complete with movements that encompassed each finger. This database consists of 15 finger movements, both individual and combined. Data were acquired in line with the standard protocol described in [25,32,33] and summarized next. 

#### 2.1.1. Electrode Application Protocol

**Figure 1:**
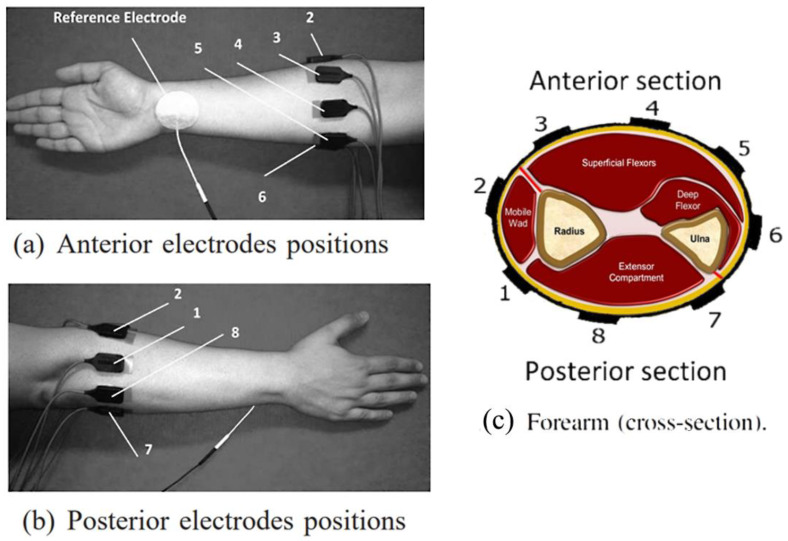
Electrode placement on the right forearm. (**a**) Anterior electrode positions; (**b**) posterior electrode positions; (**c**) muscle zones and electrodes placed on the cross-section of the forearm. (**a**,**b**) are reprinted/adapted with permission from Ref. [32], Copyright 2012, IEEE.

A ring array of eight sEMG electrodes were equally spaced across the circumference of the right forearm, with an electrode initially placed over the palmaris longus muscle, and the complete electrode set is pictured in Figure 1a,b. According to the electrode position pictures and the given reference descriptions, an illustration that approximates the distribution of the electrodes on the volume of the forearm is shown in Figure 1c. The datasets were recorded using the Bagnoli desktop EMG system (Delsys, Inc., Boston, MA, USA) [34] with the DE-2.1 sensor with 10.0 × 1.0 mm contact dimensions and 10 mm contact spacing, in differential detection mode, with an overall noise of ≤1.2 uV (RMS, R.T.I), and a bandwidth of 20–450 Hz. A 2-slot adhesive skin interface was applied on each of the sensors to firmly stick them to the skin. A conductive adhesive reference electrode (Dermatrode reference electrode) was placed on the wrist of each of the subjects during the experiments. The collected EMG signals were amplified using a total gain of 1000. A 12-bit analog-to-digital converter (National Instruments, BNC-2090) was used to sample the signal at 4000 Hz; the signal data were then acquired using Delsys EMGWorks Acquisition software. The EMG signals were then bandpass filtered between 20 and 450 Hz with a notch filter implemented to remove the 50 Hz line interference. 

#### 2.1.2. Subjects

Eight normal subjects (six males and two females) aged between 20 and 35 years were recruited to perform the required finger movements, and the execution of each movement was repeated 12 times. They were all from Sydney, with a random sampling; i.e., no specific sampling requirements were reported. The subjects were all normally limbed with no neurological or muscular disorders. All participants provided informed consent prior to participating in the study as was approved by the university research ethics committee and consented to their data being used for research purposes [25,32].

#### 2.1.3. Database Protocol

Subjects were seated on an armchair, with their arms supported and fixed at one position. The 15 movements correspond to flexions, including the flexion of each individual finger and combinations between them: thumb (T_T); index (I_I); middle (M_M); ring (R_R); little finger (L_L); the combinations T_I, T_M, T_R, T_L, I_M, M_R, R_L, I_M_R, and M_R_L; and the closed hand (HC). Table 1 indicates the class label assigned to each of the movement types in the database. The measured region has a signal detection volume that is assumed to lie between the largest number of muscular bodies and the region of the joints of the elbow; the relevance of the use of an array around the circumference of the forearm is because it also applies to trans-radial amputees [33]. In this ring electrode arrangement, the information related to the generated movement is found in the surrounding tissue and is transmitted in all directions by that section of the forearm. Although it is not possible to indicate a specific muscle for each electrode of the ring, all the electrodes pick up signals from all the muscle distribution layers. The information available is that there were electrodes in a given position when the database was captured; repeatability is likely to be acceptable as long as the same experimental protocol conditions are maintained in every test subject.

### 2.2. Raw Data, Elimination of Outliers, Windowing and Preparation of Feature Matrix

The database consists of 8 subjects and 15 movements, in which each subject performs 12 repetitions for each movement; that is, the database contains 1440 repetitions of some type of movement. A repetition of a movement is equivalent to collecting, at a rate of 4000 samples per second, the signals of eight sEMGs for 5 s. An sEMG is encoded with 12 bits, but it is read as a floating-point unit. Therefore, a repetition of a movement is equivalent to (4000 samples/s) × (5 s) = 20,000 data sample packages, including eight electrode signal channels at each sample time.

The database was downloaded from [25], where a zip file can be obtained with eight folders, one folder for each subject. Within each folder are files in CSV format named with the name of the movements. The data were loaded with MATLAB’s *csvread* function. For each subject, a matrix was created for each movement, named with the abbreviation of the movement. These matrices comprised eight sEMG columns and 240,000 rows of data, which represent the total of 12 repetitions of a movement per subject. In addition, a column with a class label number was added that identifies the type of movement, according to Table 1, so that at all times it is known which sample corresponds to each subject, type of movement and sEMG electrode number. 

**Figure 2:**
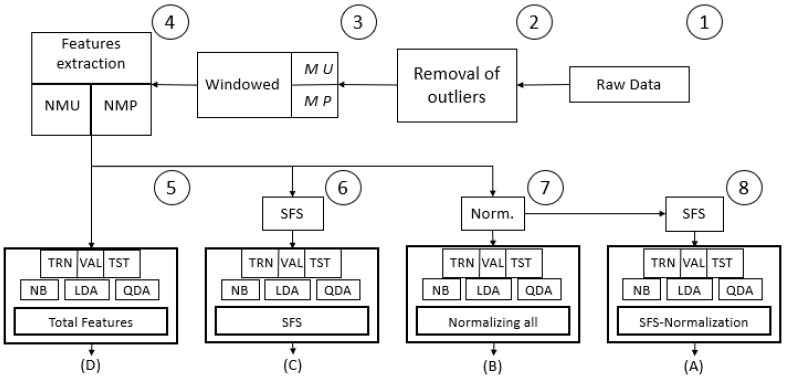
Experimentation conducted to determine the best option for creating a classification model considering recognition percentage and simplicity of the model. MU = single matrix (all eight subjects concatenated), MP = matrix per subject (eight matrices are formed). The number of circles defines the order of processing. The nomenclature (**A**–**D**) serves for Duncan’s significance test treatment identification that compares the output of each experiment.

A folder was created in MATLAB for each subject in which the 15 matrices of the movements of each subject were stored. The 15 matrices of a subject were concatenated into a new matrix named sub(i). This matrix was 3,600,000 rows by 9 columns, and it was saved for each subject. Then, in a global matrix, the data of the eight subjects were stored, forming a matrix of 28,800,000 rows and 9 columns, all of which are indicated in Figure 2 with circle 1. It should be clarified at this point that the data are ordered from subject 1 to subject 8; that is, the data in the matrix start with movements 1–15 of subject 1, then include movements 1–15 of subject 2, and so on until the movements of subject 8. When in a consecutive row, there is a change in column 9, of movement type, from label class 15 to label class 1, indicating the data edge that exists between two subjects.

Due to the nature of the signal and the large number of data, these were preprocessed with cleanup and reduction methods to concentrate the information before creating classifiers. This preprocessing included the steps of removing outliers, grouping clean data into a single global matrix and across multiple matrices (by individual subjects), setting up a data window to extract information, and measuring features per window to characterize the EMG signals.

#### 2.2.1. Method of Elimination of Outliers by a Chi-Square Distribution

According to the literature, an outlier is an observation that deviates from other observations and raises suspicions about being generated by mechanisms external to natural ones. Some statistical methods for the detection of multivariate outliers use the data distribution center and an established distance; in our case, the Mahalanobis distance was used to determine which data are outliers. The outlier elimination method with a chi-square distribution was also used [35]. The global matrix, composed of the eight subjects, was used to find outliers in the entire database. During the outlier elimination, when an irregular reading of an EMG electrode was detected, the entire line with all its eight EMG signal channels was eliminated, when the channel corresponding to that atypical event was included. This method was implemented in MATLAB, delivering a global matrix without outliers; the pseudocode for this process is shown in Algorithm 1. From this updated matrix, a separation into new matrices by individual subjects was carried out manually, and eight matrices with different lengths were obtained given the nature of the elimination of outliers. As a result of this process, the information regarding the data values eliminated and those maintained was known, as the sum of both must coincide with the total data.
**Algorithm 1** Pseudocode for Outlier RemovalThe data to evaluate are imported.Statistical parameters (Mean, Covariance) are calculated.Calculation of the parameters (New_Data, inverse covariance) to obtain the Mahalanobis distance.-Calculation of New_Data = data minus the mean.-Inverse covariance is calculated.Product of New_Data and inverse covariance.Critical value is obtained according to a chi-square distribution with the function *ncx2inv* with MATLAB.Data are separated according to the critical value.Outliers are removed and maintained matrices are created.

#### 2.2.2. Preparation of Matrices and Transformation of Data into Functional Features

With the data no longer containing outliers, we created two datasets: The first was a global matrix updated with the EMG data of all the subjects, which is called MU and has 26,778,435 data packets; 7.02% of data were eliminated for being atypical elements. The second was a dataset including the data of each subject separately, in eight matrices (called MPs), with lengths between a minimum and a maximum of 3,147,328 and 3,540,137 data packets per subject.

Taking a group of 250 rows to form a measurement window, the data were transformed into functional features. Of the 250 rows of data in the window, 12 features were calculated for each EMG channel (see Section 2.3.1), which were concatenated horizontally, resulting in a single vector of 96 electrode features for each window. In addition, the class of the motion type, to which the new vector belongs, was added in column 97. This vector of features was stored in a new matrix, continuing with each window, updating the matrix of features until reaching the end of a movement type. The final window of a specific motion type was calculated with the remaining data, which can range from 1 to 250 data rows.

The arrays have a column with the class label, and depending on the class, the data can be separated into motion subarrays. For any of the two groups of matrices (global or per subject), the MATLAB *find()* function was used to select the movements that belong to classes 1 through 15; *find()* gives us the position in which the rows of the specific movement searched were found. A submatrix of features was created for each movement with subscript j, which went from movement 1 to movement 15. Within this cycle of measurement of features, the first submatrix was taken, that is, movement 1, and once it was finished with the movement type, it started with the next one, and so on until the 15 movements were finished. At this point, 15 submatrices of each movement with their corresponding feature vectors resulted.

**Figure 3:**
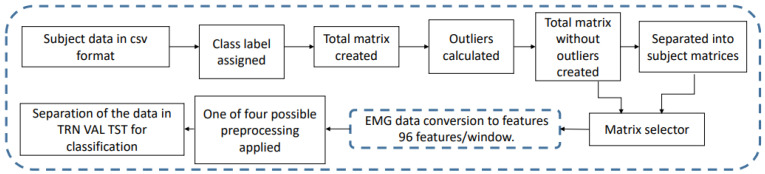
General sequence of data manipulation: from the database files to the preparation of feature matrices, before the experiments being carried out.

Upon having the features’ measurements, a rearrangement was performed to obtain the matrix of a particular subject or the one corresponding to the total MU matrix. From having the first subject, the cycle was repeated manually, adding the data of subject 2 and so on until subject 8 was reached. At the end of this process, we have the features in two types of data blocks: a matrix that groups all the subjects and their movements, named NMU, and the eight matrices that group the movements by subjects (called NMPs). These procedures are encapsulated in Figure 3.

Some investigations work only with data from individual subjects, and when modeling the classifiers by subject, very good classification percentages are obtained. Although in other studies the clustered data of a group of subjects are used to generate a single classification model applied to the people in the group, with less promising results, in this study, we compare the classifiers designed with the two types of data groups.

#### 2.2.3. Selected Features from the Literature

The literature consulted revealed that features useful for extracting information from raw EMG signals are divided into those of the time domain and those of the frequency domain, both widely used in classifiers. As one objective of this work is to generate the simplest and quickest processing in terms of execution speed, it was decided to use only features in the time domain. As mentioned in [36], one of the major disadvantages of these types of features is that a stationarity property is assumed, a property that does not coincide with the nature of the EMG signal; however, they have provided good gesture classification results in the past (see [32]).

For the size of the data windows, it is necessary to consider the processing time given to the calculation of the features. In this case, the aim is for the classification to be applied in real time; therefore, it is convenient to work with small data windows. A non-overlapping window was used to process the raw EMG data, with a length of 250 rows of raw data per window. A period of 62.5 ms is required to cover this window length since a row of raw data is captured at the sampling rate of 4 KHz.

Twelve features were proposed to be extracted from the raw EMG data, and their definitions are in [37]: mean absolute value; mean square value; simple square integral; square mean; EMG variance; TM3, TM4 and TM5 time moments; length of wave; zero crossings; myopulse percentage ratio; and curve sign change. The 12 proposed features were measured in each of the eight EMG channels and were concatenated, producing a vector of 96 data (features) for each window per subject. The windowing follows after being an MU or MP matrix, evaluating the 12 features per EMG channel to create new matrices: MU > Windowing > Features > NMU, or MP > Windowing > Features > NMPs. This data transformation process is conceptually visualized in Figure 2 and Figure 3 and was developed to go along with the classical methods in machine learning and pattern recognition to extract information from the raw EMG signal; variations can be observed in the window sizes and in the selected features.

### 2.3. Classification Models

#### 2.3.1. Machine Learning

**Figure 4:**
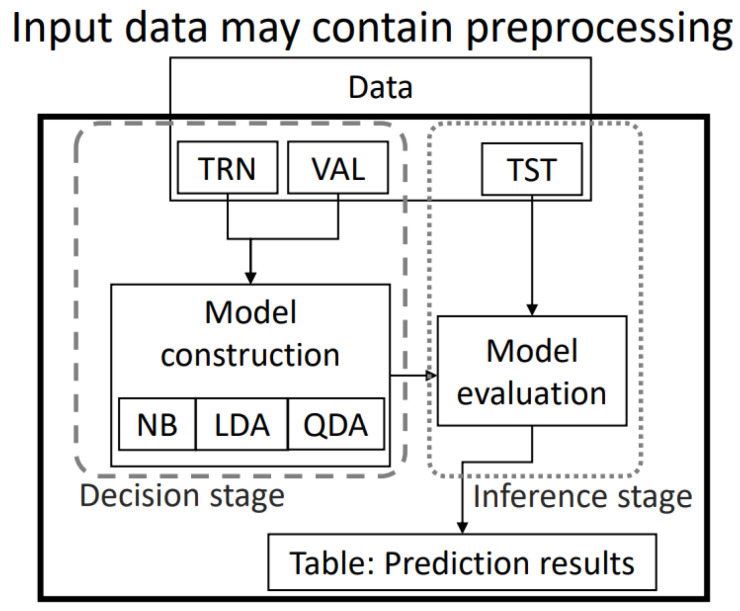
Creation of the classification model. The decision and inference stages of machine learning can be observed in the creation of classification models.

Classification problems are often divided into two stages: the decision stage and the inference stage. These two stages are visualized in Figure 4. The decision stage is when the model is being trained. The inference stage is used in classification to predict data once the model is already trained. These stages were used extensively in this study. The methodology in Figure 3 was established.

The classifiers considered for their simplicity were naive Bayes, LDA and QDA, all with a Bayesian foundation and probabilistic theory. The importance of these classifiers is that they are very efficient, as well as simple, and they have been used to implement machine learning in microcontrollers [38]. The MATLAB function *fitcdiscr* was used to create LDA and QDA, and the *fitcnb* function was used for naive Bayes.

Four classification schemes were developed, as conceptualized in Figure 2, generating combinations of two preprocessing methods; the forward sequential selection method and the normalized one, were evaluated, in addition to the version without preprocessing. The intention was to investigate the efficiency of these classifiers, comparing the different versions in case there were any statistically significant differences, and to decide the best route for conversion to a new input data space that increases the recognition of the classifier model and/or assists in reducing the number of required features.

#### 2.3.2. Cross-Validation

**Figure 5:**
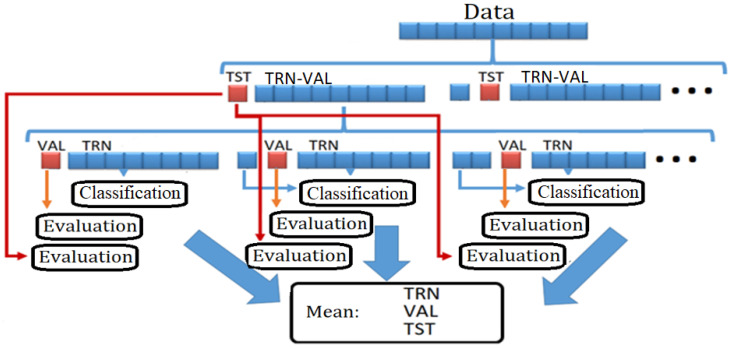
Methodology for cross-validation. The original dataset is split several times to have partitions of data into subsets for model training and evaluation.

One of the most used methods in the validation of classification models in machine learning is cross-validation. This enables, within a defined dataset, considering all data as training, validation and test data, and it validates that the results are independent of the data partitions; the calculations were based on [35]. The methodology in Figure 5 establishes the order in the separation of the data to conduct an adequate cross-validation. This process was performed for each matrix of the eight subjects and for the matrix with the data of the eight subjects (NMU). Matrices were created for training, validation and testing. A MATLAB function called *cvpartition* was used to separate the data (partition) for cross-validation. This function was used to separate the original set of data into 10 parts, 9 of which were assigned a TRN-VAL label and 1 of which was assigned a TST label. In this way, we had 10 parts of our original data. Next, the function created 10 datasets by traversing the TST label to each part of the original set (divided into 10). The function was used twice, once to separate the original set of data into 10 parts and create two blocks called TRN-VAL and TST from the original set and once to separate the data from the TRN-VAL block into two TRN sub-blocks and VAL. This process is illustrated in Figure 5: below each cvpartition block are columns of data with 10 sections, which are assigned a name. For the first block, the columns form two blocks: TRN-VAL (with nine parts) and TST (with one part). This method creates 10 datasets with the same total data. In the second cvpartition block are another 10 columns below, which is how one of the previous TRN-VAL datasets of the first block was divided. Then, 1 of those 10 sets was divided into 10 subsets of data composed of 9 parts of TRN and 1 part of VAL in each of the 10 columns.

At this point, we had 10 datasets divided into TRN-VAL and TST. In the same way, the 10 sets of TRN-VAL were now divided each into 10 new subdatasets in TRN and VAL. That is, we had 100 TRN models validated with 100 sets of VAL. Each result was averaged to calculate a real percentage of training and validation. At the end of the methodology in Figure 5, we obtained 100 classification models in which the first partition of the test (10 in total) of each dataset was tested in 10 subsets of TRN and VAL. Those results were averaged for each classifier separately.

### 2.4. Preprocessing Techniques Prior to Classification

When working with classification models obtained through machine learning, it is desirable to have features that generate maximum differentiation between classes; therefore, it is necessary to use preprocessing of features. This way, the classification models will perform better. Therefore, one task was to find the features that best separate the motion classes in their corresponding spaces and that improve prediction. A feature-selection method was used that reduces the data volume based on the quality of each feature. Another method used was the normalization of the features. Finally, the best possible combination of preprocessing was converted to a new space by the sparse matrix method.

#### 2.4.1. Sequential forward Selection (SFS)

SFS is an iterative method that provides a direct route to determine which features improve the classification results. With 12 features measured for each electrode, 96 features were generated; that is, 96 operations must be executed for each window. A large number of simple operations might work for a real-time procedure; however, the aim was to reduce the number of electrode features since some can impair the performance of the classifier.

Generally, this method analyzes each feature in an orderly manner from 1 to 96 and is an iterative process. Once the method has analyzed each feature individually, it will take the feature that provides the best percentage of recognition; the process will then form groups of two. Once it has determined the best of the individual features, the process will join this feature with each of the 95 remaining ones; this process is detailed in Algorithm 2. Then, the feature that offers the highest percentage to the classifier is selected, and so on until the percentage stagnates.
**Algorithm 2** Pseudocode Implemented for forward Sequential SelectionSPACE = 1…L                //L = number of total features.EXIT = falseWHILE NOT EXIT**FOR** i =1 **TO** |SPACE| TEMP(i) = J(SUBSET V SPACE(i))       //TEMP = Temporal variable. **END**
BEST_i = ARGMAX(TEMP)        //The maximum value of the                         //TEMP vector is stored in BEST. **IF** TEMP(BEST_i) > BEST_EVAL        //Comparison between the best stored SUBSET = [SUBSET, SPACE(BEST_i)]     //evaluation and the best evaluation BEST_EVAL = TEMP(BEST_i)         //of the new subset. SPACE = SPACE-SPACE(BEST_i)**ELSE** OUTPUT = true**END****END****RETURN** SUBSET, BEST_EVAL

Each classifier considered seeks to separate the classes in its own way, and therefore, it can take the features that offer the best results. All the features’ data were taken from each of the subjects and from the NMU matrix, and a classifier was generated for each dataset and for each model (NB, LDA and QDA). That is, the percentage of training was calculated, and the groups of features were saved based on this. By having the groups of features for each classifier considered the best, the data were taken again and separated into TRN, VAL and TST. With 10 × 10 cross-validation, a group of models was generated for each classifier. It is evident how many features remained and what percentage of classification was obtained for TRN, VAL and TST. A more detailed description can be found in [39].

#### 2.4.2. Normalization

If the set of measured features is transformed into a normal dataset (i.e., it has a zero mean and a standard deviation of 1), all the measurements of all the evaluated features are put in the same range of values. Therefore, a feature in a classifier could operate more efficiently. The procedure was conducted in a general way for the three classifier models. The description of this data transformation can be found in [35].

#### 2.4.3. Conversion to Sparse Matrix Space

**Figure 6:**
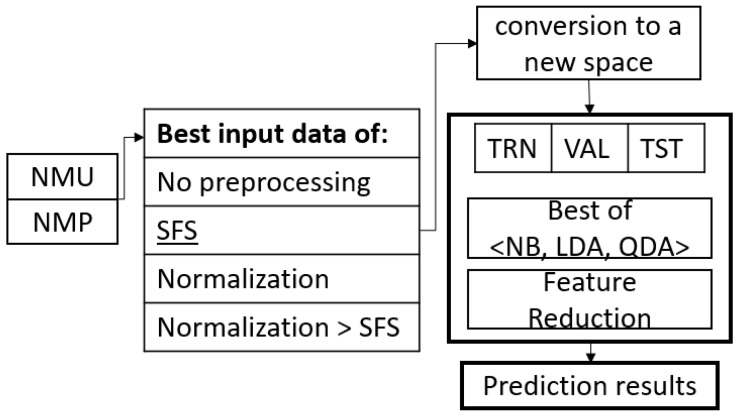
Creation of the classification model after selection of the best preprocessing from the conversion into a new space.

The conversion of features into new spaces, although it increases the processing load, can provide good results regarding increasing the recognition of the classifier model and/or reducing the number of features. It was decided to use this method because it is an innovative method that has not been widely used and its performance on muscle signals has not been reported. This algorithm is used in the first instance to increase the distances between the evaluated classes and reduce the distances between the two data of the same class. The description of this method is in [40]. The method was carried out only with the preprocessed data that provided the best results; the group of features that increased performance was taken, and these were transformed into a new space, to apply later only the best efficiency classifier model and to calculate the recognition percentage. Figure 6 illustrates the concept of conversion into a new space to improve the classification model.

### 2.5. Methods of Statistical Validation of the Results

Guided by the statistical tests from similar investigations, the proposed schemes of ANOVA method, multiple comparison test of Duncan and statistical formulations are used below for the comparison of results.

#### 2.5.1. ANOVA Method

The ANOVA method was used to compare and study the effect of the methods applied to our data regarding the means of the recognition percentages obtained from the evaluated classifiers. This analysis is a statistical test to use when comparing the means of two or more groups. The null hypothesis, from which the different types of ANOVA start, is that the means of the groups are statistically equal; that is, the mean is the same in the different groups.

#### 2.5.2. Duncan’s Method

This method is a multiple comparison test that enables us to compare the means of the treatments (procedures applied to the data and resulting in a percentage of recognition) after having rejected the null hypothesis.

#### 2.5.3. Statistical Formulations

The sensitivity, specificity, precision and F1 score are statistical parameters that enable us to evaluate the results obtained. These parameters are visualized in Table 2. They can also be obtained via a confusion matrix, in which a class of movement is compared against the other classes of movement, specifically locating each error that occurred when classifying the data. These parameters should be close to 100% for the model to be ideal.

### 2.6. Opensim Model of the Wrist and Hand Used for the Analysis

**Figure 7:**
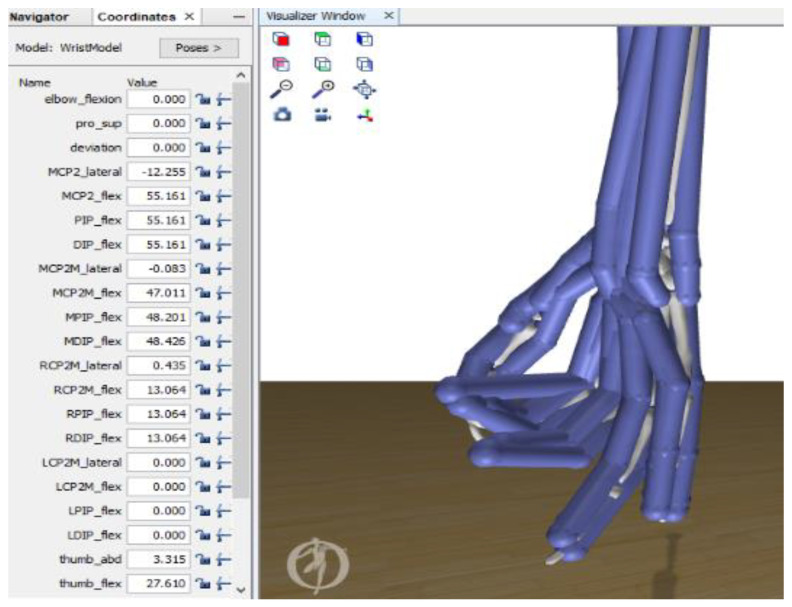
Screenshot of the software application displaying the degrees of freedom of the model used in Opensim.

Opensim software is an open access biomechanical simulation program. Among its characteristics, it enables muscle evaluation and movement analysis with visualization files with a MOT extension. In Opensim’s repository of models, there is a limited model of the wrist with 10 degrees of freedom, 28 joints and 23 actuators (muscles) with movement in the forearm, wrist, thumb (no flexion) and index [41]. This original model was modified for this study by adding the missing degrees of freedom in the thumb, middle finger, ring finger and little finger to reproduce the total movements that a human hand can perform. Figure 7 shows how the hand model appears in the graphical interface of the Opensim version 4.0 main screen.

The completed model was used for the simulation of the hand movements performed in the analyzed database. This model is found in free form in [30] and has 21 degrees of freedom and 36 joints, allowing any possible hand movement to be carried out. Table 3 lists the relationship of the degrees of freedom of the model with the movements considered from the database.

#### Simulation of Movement in Opensim

The movements were idealized for the simulation of the real movement trajectory; these must be stable and smooth in their execution. We consider that the 15 movements of the database are of this type, and each represents a complete cycle (or repetition) recorded by means of sEMG signals and described by photographs. The information on the database was used, the photographs included, but there were no other spatial measurements provided. With the EMG signal classifiers designed here, the type of movement that an input data window brings is estimated. With the predictions of the type of movement and its window time, a reproduction of the movement can be created virtually through Opensim. Every 62.5 ms (the duration of a data window), an output generated in the classifier demands the next video frame for the virtual model in Opensim. For this reason, advancement vectors were created for each of the movement classes, which contain the degree of rotation of each joint involved capable of reproducing any of the movements in the database. 

In principle, we deduce the 15 ideal movements that the database contains. Therefore, the initial and final positions of the movements were taken as a reference based on their description [32]. Considering that each movement lasts for 5 s and that the number of decisions of the classifier in that period is 80 window times, and with the full range of motion of each of the involved joints, their degree of rotation was calculated as the progress of the movement video frame. Thus, the 15 advance vectors were formed for each movement class; Table 4 lists all of them. Then, after obtaining the tag predictions of the classifier and having the table of advance vectors, a motion file can be created for any complete finger movement input, as shown in Algorithm 3. In addition, 15 ideal motion files with the MOT extension can be created by simply accumulating the specific advancement vector itself 80 times, corresponding to the 5 s of a movement repetition. Once the MOT files are obtained, any movements can be viewed using the Opensim platform.
**Algorithm 3** Pseudocode for the Creation of a Motion File From Classification. MOV_NUMBER Matrix Corresponds to Advancement Vector Table 4MOV_MATRIX = [zeros]count = 0**FOR** i **TO** |MAX Window number|Decision = Model Decision(i)**IF** Decision **is** 1**if** count < 18MOV_MATRIX(i + 1,:) = MOV_MATRIX(i,:) + MOV_NUMBER(1,:)count = count + 1**else**MOV_MATRIX(i + 1,:)= MOV_MATRIX(i,:) + MOV_NUMBER(2,:)**ELSE**MOV_MATRIX(i + 1,:)= MOV_MATRIX(i,:) + MOV_NUMBER(Decision + 1,:)**END****RETURN** MOV_MATRIX

It is possible to observe the movements characterized by classification errors. The visualization of a badly classified movement can offer a glimpse of how serious or acceptable that error may be; it is simply a tool to discern a movement classification. The test data can be separated to verify which of the subjects has a worse ranking. This process identifies the worst movement evaluated to appreciate a real reproduction (with complete data) of a subject´s repetition movement in the simulation.

## 3. Results

### 3.1. Preprocessing and Processing of the EMG Signal

#### 3.1.1. Elimination of Outliers

**Figure 8:**
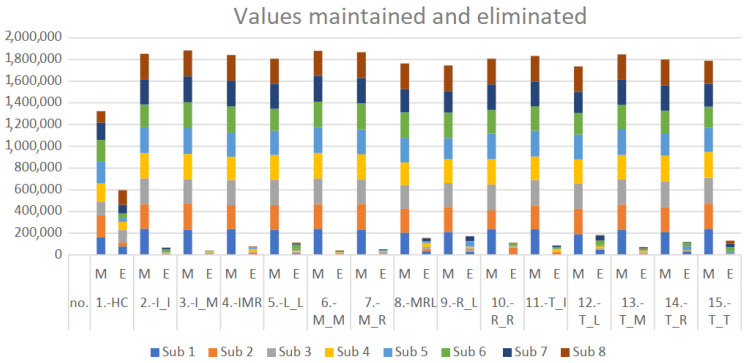
Outliers maintained and eliminated. M = maintained, E = eliminated. The total data for the 8 subjects (sub #, subject number) in the 15 movements are displayed.

The total raw data for each movement were 240,000 lines per subject. However, due to eliminating outliers, these data were reduced, generating a number of data maintained per movement and per subject. The percentage data eliminated by movements ranged from 1.99% (in I_M) to 26.00% (in HC). The percentages of the data eliminated per subject ranged from 1.66% (subject 5) to 12.57% (subject 4). Figure 8 is a graph encompassing the maintained and eliminated values, with information on movement and subject, illustrating what remains of the raw data per movement and per subject.

#### 3.1.2. Feature Selection

After removing the outliers from the total set of raw data, windows of 250 data rows were created in the resulting matrix, in which each row has the digitized signals of the eight sEMG channels. In each window, 12 features were measured for each sEMG channel, according to the processes in Section 2.2.3, resulting in 96 electrode features per window. Then, the SFS algorithm evaluated the performance of each feature in each of the considered electrodes.

The SFS algorithm was applied in two scenarios, one scenario using the features in their originally measured range and the other scenario using the normalized features, which are the experiments marked 6 and 8, respectively, in Figure 2. 

#### 3.1.3. Classification Models 

The results regarding the classification models generated the Table 5, Table 6, Table 7 and Table 8, which are displayed according to the experimentation set out in Figure 2, carried out to determine which is the best option for creating a classification model considering the recognition percentage. The experimentation was conducted using a 10 × 10 cross-validation of TRN, VAL and TST for each classifier generated from NB, LDA and QDA. Table 5 lists the recognition percentages when all 96 features were used without preprocessing. Table 6 lists the number of features obtained from the SFS algorithm and the percentage of recognition obtained. In Table 6, with all 96 features, the best classifier created was LDA; however, in Table 6, with the selected features, the best classifier is QDA, which improves significantly and has a lower number of features than those initially considered.

Table 7 contains the results of the classification with the set of 96 features used but normalized. The worst classifier is NB, and the best classifier belongs to QDA. Subsequently, in Table 8, the best normalized features are selected. The worst classifier continues to be NB, and the best QDA, both for individual subjects and for the sample group. Although NB has a smaller number of necessary features, the difference between the recognition percentages is considerable. The total of normalized features for Table 7 is 96, and in Table 8 the total of necessary normalized features is reduced but depends on each classifier with SFS.

The effect that the normalization of the data produces is evident in Table 5 and Table 7 without SFS, using the 96 features. The normalization effect significantly improves the recognition percentage of the QDA. However, as shown in Table 6 and Table 8, when the electrode features are selected, the normalization only produces a slight increase in the recognition percentage of QDA, which had already been improved with the pure selection of features without normalization. From Table 6 and Table 8, it is evident how many features-electrodes can be discarded without affecting the percentage of classification obtained for TRN, VAL and TST.

### 3.2. Statistical Parameters, ANOVA and Duncan Method

The results in Table 5, Table 6, Table 7 and Table 8, regarding the performance of the four treatments outlined in Figure 2, indicate not only the good results of QDA, but also that the selection of features and normalization is an important factor in the performance of this type of classifier. An advantageous difference is that SFS reduces the number of features considered.

Among the tests carried out was an inquiry with subsequent statistical validation trials regarding which of the four treatments are significant. Therefore, the eight subjects, who are part of the same population, were taken with the results of their individual QDA classifier. The four data treatments established were the following:

(A) Normalization and SFS (Table 8);
(B) Only normalization (Table 7);
(C) Only with SFS (Table 6);
(D) Without any processing (Table 5).

Table 9 lists the averages of the 10 × 10 cross-validation of the TST data subset of each treatment of each subject. The ANOVA test performed exhibited a significant difference between the means of the results (alpha = 0.05, F = 34.37, *p*-value < 0.00001), rejecting the null hypothesis.

Duncan’s method was applied to determine which of the treatments are statistically the same and which are different. As shown in Table 10, Duncan’s method revealed that µA = µC, µA = µB, µC = µB, µC ≠ µD, µC ≠ µD and µB ≠ µD; therefore, treatments A, B and C are statistically the same and D is different. Therefore, we can select any of A, B and C. However, as A provides us with a low number of features and a higher value in the recognition percentage, it was decided to use QDA with normalization and SFS to continue working with these conditions.

### 3.3. Conversion to a New Sparse Matrix Space

**Figure 9:**
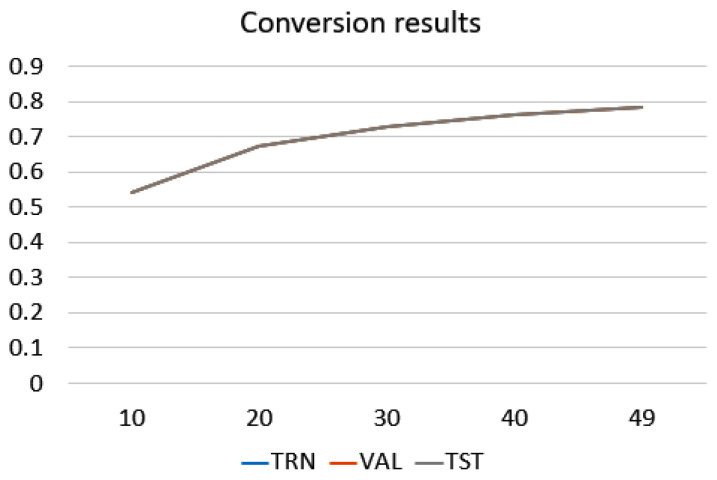
Plot: percentage of classification by selecting a smaller number of features after conversion to sparse matrices.

As established, this method was used to reduce further the number of features used, to increase the percentage of model recognition and to generate a simpler model. Figure 9 illustrates the main features versus the recognition curve. The more main features are input, the more the percentage of recognition grows, but it was only possible to reach the highest classification rate as before.

### 3.4. Statistical Formulations for QDA after Space Change

**Figure 10:**
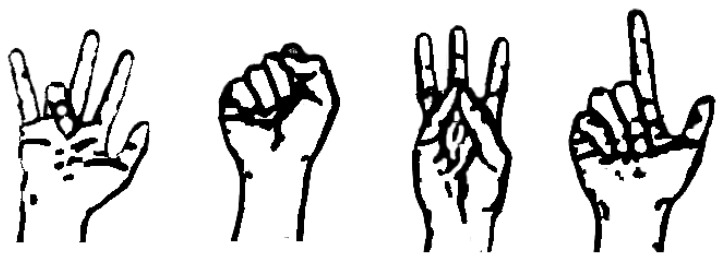
Hand gestures with perfect recognition. From left to right, the classes 10 (R_R), 1 (HC), 12 (T_L) and 8 (MRL).

The statistical parameters considered—sensitivity, specificity, precision and F1 score—for each of the 15 classes were calculated. Table 11 presents these parameters for the average of the eight subjects evaluated individually; data for the group sample of the eight subjects are presented in Table 12. As shown in Table 11, the classes 1 (HC), 8 (MRL), 10 (R_R) and 12 (T_L) are movements that have an ideal classification; Figure 10 illustrates these hand gestures.

### 3.5. Evaluation of the Movement in Opensim

**Figure 11:**
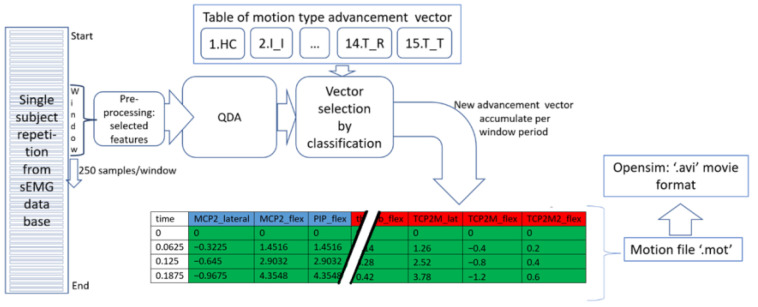
Cycle from classification to generation of the movement file for Opensim.

Each new classification of a data window is translated into an advancement vector, which integrates the joints involved in the movement detected, representing a progress proportional to 62.5 ms of a total motion duration of 5 s. Figure 11 visualizes the complete cycle, from the detection of the movement with the classifier to the choice of the corresponding advance vector and the addition of this vector to the movement matrix. Once the movement has finished, the motion file with the MOT extension was used for the subsequent motion visualization in Opensim. With the generated movement files, we performed biomechanical movement analysis in Opensim, verifying the activations and muscular participation within the movements. Some ideal motion files and their videos are in [30]. 

**Figure 12:**
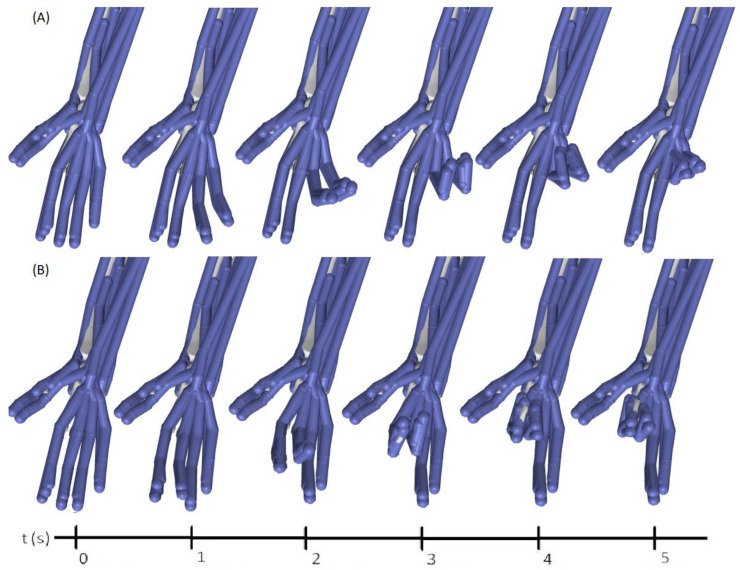
Reproduction of the RL movement for 5 s in Opensim: (**A**) ideal movement; (**B**) total movement of repetition 1 for the movement of subject 6, which was the worst classified.

Evaluating the movement classified with an Opensim visualization is a way of illustrating the performance of the classifier. After analyzing the recognition of each subject and its test data class label (type of movement), it was found that, on average, the worst classified is the RL movement, with subject 6 having the worst recognition percentage for this movement. Figure 12A illustrates the Opensim reproduction of the ideal RL movement for 5 s. Figure 12B visualizes the classification of the complete repetition of the RL movement of subject 6 (repetition 1 was taken). A complete move can take up to a maximum of 80 classification events if there are no outliers removed that reduced the number of windows. In this repetition of RL, only 76 classifications were provided, of which 11 were erroneous for the RM movement (14.5%) and the others were for the IM movement (85.5%). A predominance of the middle finger was observed in the misclassification of movement. In Figure 12B, it is evident how the middle finger progresses most before any other. How serious the misclassification can be depends on the task to be performed and the application.

## 4. Discussion

The objective of this work was the evaluation of an open access electromyography database for the detection of 15 movements of the fingers of the hand through a simple preprocessing-enhanced classifier to visualize the muscular participation of the predicted movements in the virtual environment of Opensim and generate new insight for prosthetic application. The usefulness of the Opensim musculoskeletal simulation environment in the design of a multifunctional prosthesis was demonstrated, and the evolution of muscle movements was virtually observed from the results of the classification model and visually compared with the ideal movement. Working with multiple specific movements (the flexion of each finger and combinations of flexions) from a database provided a multifunctional complex system outside of laboratory environments, where the control of upper-limb prosthetic systems can be investigated in a practical way. The standard models of classification implemented with a short data window provided a good recognition rate (in accuracy and number of classes) and will be easy to embed into microcontrollers to classify a sequence of movements in real time in the future. A description of specific results follows, along with a discussion of the limitations of the study.

The method for outlier elimination indicates that the closed hand movement was the most affected, as seen in Figure 9. This was the most complex hand gesture because it involved flexing all the fingers, which would have favored the proliferation of their outliers. However, the closed hand flexion is one of the most common movements in daily life, and, at the same time, it was one of the best-classified movements. 

After the removal of outliers, without any type of preprocessing, of the four classifiers created in Table 5 (experimentation 5 in Figure 2), the best of the algorithms is LDA for the two groups of data matrices. Table 6 presents the results for when the features were selected in the creation of the classifier. One more column is displayed, in which the number of features selected in each classification algorithm is defined. With a set of features selected from the total set, the classification percentages increase, with the most notable change being in QDA, reaching 77.86% recognition of the global subject matrix and up to 94.73% with the individual matrices of the subjects. 

The next set of results is shown in Table 7 and Table 8, in which the effects of normalization are evidenced in both cases, either as a single preprocessing or in conjunction with the selection of features. It is clear there is an improvement in all the algorithms, but again the QDA model is especially improved. Selecting the features or making none predominate in the magnitude of their effect helps the classifier. In Table 8, QDA has the best performance for the 15 movements, with 96.15% recognition for subjects individually and 78.36% recognition for the sample group with the eight subjects together. 

For the input vector with 96 initial features, these features can be reduced without degrading the recognition; and with selected features, they even improve the recognition of the classifiers. In QDA, 25 electrode features remained, on average, for individual subjects, and 49 remained for the sample group. A curious fact about the electrode features selected and the position of the significant electrodes is that QDA achieved its classification rate using practically only four electrodes. Electrodes 3, 4, 5 and 6 were the ones that behaved the best and correspond to being better positioned to capture the signal from the active flexor muscles, according to Figure 1. This point demonstrates how redundant the EMG electrode ring array setup can become. As revealed by the SFS tests on the NB and QDA classifiers, there is potential to tailor the methodology specifically with a few electrode features to an individual (up to only 17 in a subject) and achieve a good classification percentage.

The results of each procedure performed are supported by the cross-validation included in them and even by an ANOVA test. When the results obtained from the experimental treatments were evaluated with Duncan’s multiple comparisons test, the latter three experimental procedures (marked 6, 7 and 8 in Figure 2 and Table 6, Table 7 and Table 8) were similar; that is, the means of the recognition percentage measurements were not statistically different. Therefore, we could use any of those three treatments; however, for the moment, to continue investigating, the chosen model was the quadratic model with a selection of normalized features. 

The statistical formulations of sensitivity, specificity, precision and F1 score of the eight subjects evaluated separately (Table 11) reveal that the movements 1 HC, 8 MRL, 10 R and 12 TL all have a value of 100% recognition; Figure 11 illustrates these hand gestures. This finding indicates that the QDA classifier trained for any subject could distinguish these four gestures without any error. However, a perfect class recognition does not happen when the data of the eight subjects are assembled into a single data group and processed to generate a single model for all subjects (Table 12), as is often the case. In this group, a large specificity is maintained for all classes, and the F1 score indicates a good performance for classes 1 HC and 12 T_L again, confirming them as two robust hand gestures. The class movement 13 T_M might appear to have good performance since it had the greatest sensitivity of the group, but it had a low precision. Regarding the worst class prediction for the subjects evaluated individually (Table 11), the flexion of the index finger (2 I_I) had the lowest sensitivity and specificity, which is in agreement with other research [18], and also the thumb flexion (15 T_T) presents the lowest precision and F1 score. This might correspond to the high levels of fine motor dexterity developed between the thumb and index finger that make them difficult to individually define. As for the whole group classifier (Table 12), the F1 score is above 63%, with most classes being balanced, but class 9 (R_L) shows a loss in sensitivity and class 7 (M_R) shows a loss in precision. In these two movements, which involve the ring finger flexion and the middle finger, misclassification was observed; there were repetitions with large classification errors and mistagging between themselves.

In Figure 9, it is possible to verify that there is no convenience in converting the features into a new space through the sparse matrix method. It simply maintained the recognition percentage already present, so no improvement was seen using the method, and the increased processing load with its use in a microcontroller is not justified.

To simulate the movements in Opensim, after an output result of the classifier, an advance vector was chosen and added to the construction of the simulation; therefore, the class of the movement detected provides each joint of the biomechanical model of the hand with a possible change in the degrees of rotation. At the end of the reading of the 5 s motion, there is a virtual movement matrix representative of the classification. In this way, it was possible to reproduce the classified movements of any of the subjects, whether they were of high hits or poor performance in the classification. Several videos with the movements of all the joints are in [30,42]. With these movement files (MOT format), in Opensim, we performed movement analysis and verified muscle participation within each movement evaluated. 

In the videos of a complete movement, the wrong classification of an event does not necessarily affect the total movement generated. For example, a single error of a window period translated into movement can be expressed in the same way as a movement executed at 98%, and perhaps more if the correct movements of some joints are considered within the errors. For example, if a classification indicates flexion of the index–middle–ring fingers, and it is actually a movement of the index finger, this is a partially good classification, as this movement partially helps the correct movement. Even in a normal human hand, usually, when we want to execute a single movement, taking the ring finger as an example, the hand generates some movements in the other fingers.

There are some issues to consider in this study. Although there was no significant difference in the results of the SFS and SFS-normalized tests of the three algorithms, a further discussion is required regarding the final specifications of the system, whether to work with the minimum number of electrodes, whether to have the best recognition or whether it is a matter of real-time implementation. There is a warning related to the findings on the reduction in the numbers of electrodes and features before application; although the database used in this research is of the multifunction type, it only included flexion movements and there was never any extension of a finger. Therefore, further experimentation with all kinds of finger motions would be required to optimize a classifier. An important argument in the works involving EMG signals is the repeatability in the acquisition of these measurements [43], specifically the difficulty of electrode placement accuracy. Since data mining and machine learning analyze the information as it is acquired, a key point in obtaining more reliable classification models is ensuring the same conditions of the experimental protocol during every measurement. We made sure that the selected database complied with international best practices, although the material in the dataset used did not include information about the repeatability of the EMG measurements, and this is a limitation of the work; at any rate, statistical parameters for the reproducibility of classification were calculated as a measure of performance. Another limitation of the study was that the database did not have motion sensors or video recordings that would allow us to have a direct relationship between EMG signal time and position in space. The model of Opensim was controlled by the classification results of the machine learning algorithms; for this reason, the simulated hand movements might be different from the actual hand movements.

In the future, the validation of the Opensim model using a dataset including the motion data, such as data from a 3D motion capture system or camera, will be important to improve the results of this research and to confirm the virtually created movement with the real movement position data. In addition, another task would be to bring the 15 discriminant functions of the QDA classifier, which are a sum of multiplications, to the world of portability by embedding them in a microcontroller, which allows us to be one step closer to real-time processing.

## 5. Conclusions

The development presented combined biomechanical simulation with automatic classification of 15 finger movements. An open access database containing the signals from an array of forearm EMG electrodes was used as input, and traditional machine learning and signal preprocessing methods were used for the design of the classifiers. The result turns out to be a nice tool for the practical design of hand prostheses or for human–computer interface control through hand gestures. It allows one to visualize the result of classifying a finger movement to consider its performance.

The algorithms created and their input data preprocessing provided good results in the classification of the finger movements. The QDA algorithm with SFS and data normalization provided the highest recognition rate (96.16%). The experiments suggest it is possible to make a classifier specific to a person, using only 17 electrode features and 4 EMG electrodes. This leads us towards a possible practical implementation and portability of EMG matrix control.

## Figures and Tables

**Table 1:** Relationship of movement types of the database and their class labels assigned. Flexion of the fingers involved is indicated.

Class Label	Movements Evaluated	Description
1	HC	Closed hand
2	I_I	Index
3	I_M	Index–middle
4	IMR	Index–middle–ring
5	L_L	Little finger
6	M_M	Middle
7	M_R	Middle–ring
8	MRL	Middle–ring–little
9	R_L	Ring–little
10	R_R	Ring
11	T_I	Thumb–index
12	T_L	Thumb–little
13	T_M	Thumb–middle
14	T_R	Thumb–ring
15	T_T	Thumb

**Table 2:** Statistical formulations, in which TP = true positive, FP = false positive, TN = true negative and FN = false negative.

Formulation	Operation
A = Sensitivity	TP/(TP + FN)
B = Specificity	TN/(TN + FP)
C = Precision	TP/(TP + FP)
D = F1 score	2TP/(2TP + FP + FN)

**Table 3:** Relationship degrees of freedom (DoFs) of the Opensim model of the wrist with the movements of the database.

	Database Finger Movements														
DoF	HC	I	IM	IMR	L	M	MR	MRL	RL	L	TI	TL	TM	TR	T
MCP2_lateral	x	x	x	x							x				
MCP2_flex	x	x	x	x							x				
PIP_flex	x	x	x	x							x				
DIP_flex	x	x	x	x							x				
MCP2M_flex	x		x	x		x	x	x					x		
MPIP_flex	x		x	x		x	x	x					x		
MDIP_flex	x		x	x		x	x	x					x		
RCP2M_lateral	x			x			x	x	x					x	
RCP2M_flex	x			x			x	x	x					x	
RPIP_flex	x			x			x	x	x					x	
RDIP_flex	x			x			x	x	x					x	
LCP2M_lateral	x				x			x	x	x		x			
LCP2M_flex	x				x			x	x	x		x			
LPIP_flex	x				x			x	x	x		x			
LDIP_flex	x				x			x	x	x		x			
thumb_abd	x										x	x	x	x	x
thumb_flex	x										x	x	x	x	x
TCP2M_lateral	x										x	x	x	x	x
TCP2M_flex	x										x	x	x	x	x
TCP2M2_flex	x										x	x	x	x	x

**Table 4:** Advancement vectors for each of the movement classes. A vector contains the degrees of rotation for each of the joints considered in the movement. A special case is the hand close motion, which required one of two possible vectors, depending on the time position of the motion. The labels of the joints correspond to the Opensim model [30].

	Joints/DoFs																				
	MCP2_ lateral	MCP2_ flex	PIP_ flex	DIP_ flex	MCP2M_ lateral	MCP2M_ flex	MPIP_ flex	MDIP_ flex	RCP2M_ lateral	RCP2M_ flex	RPIP_ flex	RDIP_ flex	LCP2M_ lateral	LCP2M_ flex	LPIP_ flex	LDIP_ flex	thumb_ abd	thumb_ flex	TCP2M_ lateral	TCP2M_ flex	TCP2M2 _flex
HC1	−0.3225	1.4516	1.4516	1.4516	0	1.4516	1.4516	1.4516	0.0483	1.4516	1.4516	1.4516	0.3225	1.4516	1.4516	1.4516	0	0	0	0	0
HC2	−0.3225	1.4516	1.4516	1.4516	0	1.4516	1.4516	1.4516	0.0483	1.4516	1.4516	1.4516	0.3225	1.4516	1.4516	1.4516	0.14	1.26	−0.4	0.2	0.84
I_I	−0.3225	1.4516	1.4516	1.4516	0	0	0	0	0	0	0	0	0	0	0	0	0	0	0	0	0
I_M	−0.3225	1.4516	1.4516	1.4516	0	1.4516	1.4516	1.4516	0	0	0	0	0	0	0	0	0	0	0	0	0
IMR	−0.3225	1.4516	1.4516	1.4516	0	1.4516	1.4516	1.4516	0.0483	1.4516	1.4516	1.4516	0	0	0	0	0	0	0	0	0
L_L	0	0	0	0	0	0	0	0	0	0	0	0	0.3225	1.4516	1.4516	1.4516	0	0	0	0	0
M_M	0	0	0	0	0	1.4516	1.4516	1.4516	0	0	0	0	0	0	0	0	0	0	0	0	0
M_R	0	0	0	0	0	1.4516	1.4516	1.4516	0.0483	1.4516	1.4516	1.4516	0	0	0	0	0	0	0	0	0
MRL	0	0	0	0	0	1.4516	1.4516	1.4516	0.0483	1.4516	1.4516	1.4516	0.3225	1.4516	1.4516	1.4516	0	0	0	0	0
R_L	0	0	0	0	0	0	0	0	0.0483	1.4516	1.4516	1.4516	0.3225	1.4516	1.4516	1.4516	0	0	0	0	0
R_R	0	0	0	0	0	0	0	0	0.0483	1.4516	1.4516	1.4516	0	0	0	0	0	0	0	0	0
T_I	0	0.3625	0.675	0.75	0	0	0	0	0	0	0	0	0	0	0	0	0	0.7875	−0.25	0.2875	0.1273
T_L	0	0	0	0	0	0	0	0	0	0	0	0	0	0.0585	0.95	0.8812	0.175	1.125	−0.25	0.4125	0.1825
T_M	0	0	0	0	−0.0412	0.28	0.875	0.9875	0	0	0	0	0	0	0	0	0.1875	0.575	−0.25	0.3825	0.325
T_R	0	0	0	0	0	0	0	0	0	0.1691	0.9875	0.875	0	0	0	0	0.1812	0.875	−0.25	0.3678	0.3125
T_T	0	0	0	0	0	0	0	0	0	0	0	0	0	0	0	0	0.14	1.26	−0.4	0.2	0.84

**Table 5:** Experimentation 5: Percentage of recognition using a 10 × 10 cross-validation by dividing the data into training (TRN), validation (VAL) and test (TST). The subject column is divided into two parts for each classifier; 1…8 indicates that an average was performed between the data of the eight subjects; S1:8 indicates that the total data were a single set of the eight individuals.

Experimentation No. 5 without SFS				
	Sub	TRN	VAL	TST
NB	1…8	89.38	89.38	89.38
	S1:8	29.3	29.3	29.3
LDA	1…8	93.22	93.21	93.23
	S1:8	53	52.99	52.96
QDA	1…8	67.5	68.1	68.01
	S1:8	33.43	29.84	29.79

**Table 6:** Experimentation 6: Percentage of recognition using a 10 × 10 cross-validation by dividing the data into training (TRN), validation (VAL) and test (TST). The subject column is divided into two parts for each classifier; 1…8 indicates that an average was performed between the data of the eight subjects; S1:8 indicates that the total data were a single set of the eight individuals. The N.F column is the number of features selected through SFS.

Experimentation No. 6 with SFS					
	Sub	N.F	TRN	VAL	TST
NB	1…8	21	90.71	90.72	90.72
	S1:8	27	39.15	39.16	39.18
LDA	1…8	57	93.08	93.10	93.11
	S1:8	80	53.18	53.19	53.15
QDA	1…8	30	94.68	94.72	94.73
	S1:8	24	77.71	77.88	77.86

**Table 7:** Experimentation 7: The format of the table is the same as Table 5, with the total number of features used in the tests; normalization was applied to the data before they were used to create the model.

Experimentation No. 7 without SFS				
	Sub	TRN	VAL	TST
NB	1…8	89.94	89.97	89.96
	S1:8	29.31	29.3	29.30
LDA	1…8	93.6	93.61	93.60
	S1:8	54.79	54.8	54.78
QDA	1…8	94.06	94.06	94.05
	S1:8	51.15	51.04	51.07

**Table 8:** Experimentation 8: The format of the table is the same as Table 6, with the number of features selected for each subset of tests from SFS; normalization was applied to the data before they were used to create the model.

Experimentation N. 8 Normalization with SFS					
	Sub	N.F	TRN	VAL	TST
**NB**	**1…8**	21	91.33	91.36	91.34
	**S1:8**	27	39.15	39.16	39.18
**LDA**	**1…8**	40	93.10	93.12	93.12
	**S1:8**	69	54.73	54.74	54.75
**QDA**	**1…8**	25	96.09	96.14	96.16
	**S1:8**	49	78.34	78.36	78.36

**Table 9:** Percentage of recognition for each treatment for each subject with QDA.

Treatments				
Subjects	Without Any Processing	Only with SFS	Only Normalization	Normalization and SFS
**1**	65.97	96.53	96.68	98.27
**2**	88.82	95.38	96.37	97.76
**3**	59.06	91.80	87.57	91.87
**4**	71.41	92.26	92.32	93.93
**5**	73.89	97.06	97.48	98.94
**6**	72.09	95.47	96.92	97.44
**7**	65.46	95.35	96.06	97.06
**8**	47.36	94.01	89.04	93.98
**Average**	68.01	94.73	94.01	96.16

**Table 10:** Table for results of Duncan’s significance testing; the conclusion is that the means of the results obtained from the procedures A, B and C are equal, and D (without any processing) is not equal. Therefore, any of the treatments “normalization and SFS”, “only normalization” and “only with SFS” can be chosen.

Population Difference	Sample Difference Compared to Their Rp Range	Decision
${µ}_{A}-{µ}_{C}$	96.15–94.73 = 1.42 < 7.1 = $R_{4}$	Not significant
${µ}_{A}-{µ}_{B}$	96.15–94.05 = 2.10 < 7.0 = $R_{3}$	Not significant
${µ}_{A}-{µ}_{D}$	96.15–68 = 28.14 > 6.6 = $R_{2}$	Significant
${µ}_{C}-{µ}_{B}$	94.73–94 = 0.67 < 7.0 = $R_{3}$	Not significant
${µ}_{C}-{µ}_{D}$	94.73–68 = 26.75 > 6.6 = $R_{2}$	Significant
${µ}_{B}-{µ}_{D}$	94–68 = 26.04 > 6.6 = $R_{2}$	Significant

**Table 11:** Average of the statistical parameters for the eight subjects evaluated individually.

QDA				
Class	Sensitivity	Specificity	Precision	F1 Score
1	100	100	100	100
2	88.65	99.09	88.65	88.65
3	96.90	99.74	96.90	96.90
4	98.88	99.83	97.80	98.34
5	98.68	100	100	99.33
6	97.32	99.91	99.09	98.19
7	97.80	99.91	98.88	98.34
8	100	100	100	100
9	97.40	99.42	91.46	94.33
10	100	100	100	100
11	92.52	99.74	97.05	94.73
12	100	100	100	100
13	97.75	99.50	93.54	95.60
14	92.39	99.83	97.70	94.97
15	90.78	99.10	86.25	88.46

**Table 12:** Statistical parameters for the dataset formed by the group of eight subjects with 49 electrode features.

QDA				
Class	Sensitivity	Specificity	Precision	F1 Score
1	83.26	99.78	97.11	89.65
2	78.99	98.09	78.77	78.88
3	81.87	96.74	70.18	75.57
4	70.71	98.06	77.05	73.75
5	70.05	98.87	85.00	76.80
6	79.25	96.81	68.87	73.69
7	86.20	94.82	58.36	69.60
8	83.33	98.42	81.63	82.47
9	52.60	98.76	78.73	63.06
10	69.56	98.77	82.90	75.65
11	87.71	98.15	80.85	84.14
12	79.65	99.78	97.25	87.58
13	93.19	97.81	79.06	85.54
14	84.44	98.17	81.02	82.69
15	77.91	98.37	81.18	79.51

## Data Availability

Publicly available datasets were analyzed in this study. This data can be found here: [https://www.rami-khushaba.com/electromyogram-emg-repository.html] (accessed on 21 March 2022), [https://simtk.org/projects/moving-fingers] (accessed on 21 March 2022).
